# Hippocampal RNA sequencing in mice selectively bred for high and low activity

**DOI:** 10.1111/gbb.12832

**Published:** 2022-12-13

**Authors:** Winona C. Booher, Lauren A. Vanderlinden, Lucy A. Hall, Aimee L. Thomas, Luke M. Evans, Laura M. Saba, Marissa A. Ehringer

**Affiliations:** ^1^ Department of Pharmaceutical Sciences, Skaggs School of Pharmacy and Pharmaceutical Sciences University of Colorado Anschutz Medical Campus Aurora Colorado USA; ^2^ Institute for Behavioral Genetics University of Colorado Boulder Boulder Colorado USA; ^3^ Department of Integrative Physiology University of Colorado Boulder Boulder Colorado USA; ^4^ Department of Biostatistics & Informatics, Colorado School of Public Health University of Colorado Anschutz Medical Campus Aurora Colorado USA

**Keywords:** anxiety, behavioral battery, enrichment analysis, hippocampus, mitochondria, open‐field activity (OFA), PANTHER, quantitative trait loci (QTL), RNA‐sequencing, wheel running

## Abstract

High and Low Activity strains of mice were bidirectionally selected for differences in open‐field activity (DeFries et al., 1978, Behavior Genetics, 8: 3–13) and subsequently inbred to use as a genetic model for studying anxiety‐like behaviors (Booher et al., 2021, Genes, Brain and Behavior, 20: e12730). Hippocampal RNA‐sequencing of the High and Low Activity mice identified 3901 differentially expressed protein‐coding genes, with both sex‐dependent and sex‐independent effects. Functional enrichment analysis (PANTHER) highlighted 15 gene ontology terms, which allowed us to create a narrow list of 264 top candidate genes. Of the top candidate genes, 46 encoded four Complexes (I, II, IV and V) and two electron carriers (cytochrome c and ubiquinone) of the mitochondrial oxidative phosphorylation process. The most striking results were in the female high anxiety, Low Activity mice, where 39/46 genes relating to oxidative phosphorylation were upregulated. In addition, comparison of our top candidate genes with two previously curated High and Low Activity gene lists highlight 24 overlapping genes, where *Ndufa13*, which encodes the supernumerary subunit A13 of complex I, was the only gene to be included in all three lists. Mitochondrial dysfunction has recently been implicated as both a cause and effect of anxiety‐related disorders and thus should be further explored as a possible novel pharmaceutical treatment for anxiety disorders.

## Graphical abstract

Overview of differentially expressed genes by strain and sex.

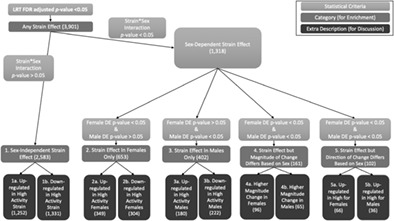

## 1 INTRODUCTION

Anxiety disorders are one of the most common mental health disorders and are approximately twice as prevalent in females.^1^, ^2^ They are estimated to affect up to 20% of adults each year, however the true prevalence is likely even greater as many people do not seek treatment or clinicians fail to make the diagnosis.^3^, ^4^ In fact, a study conducted in European countries reported that only 21% of respondents with an anxiety disorder sought professional help.^5^, ^6^ Additionally, in 2013 it was estimated that anxiety disorders constitute more than 30% of the total expenses for mental illness, totaling approximately $48.72 billion.^7^, ^8^ Thus, the daily anguish felt by those with anxiety disorders and subsequent economic burden highlights the importance of understanding the neurobiological basis of anxiety disorders.

Currently, anxiety disorders are assumed to be an interaction of psychosocial factors and a heritable vulnerability, which disrupts the brain neurotransmitter systems.^6^ Recently, accumulating evidence has implicated brain mitochondria and bioenergetics in the development of psychiatric disorders, including anxiety disorders.^9^, ^10^, ^11^, ^12^, ^13^, ^14^ In fact, patients with mitochondrial disorders frequently exhibit anxiety symptoms and key alterations in mitochondrial function have been identified in highly anxious individuals.^9^, ^13^, ^14^ In addition, increased oxidative stress has also been linked with highly anxious individuals^15^ and higher mitochondrial DNA copy number (mtDNA‐cn) has been reported in leukocytes of humans that have suffered early life stress.^13^ Furthermore, MitoQ is an over‐the‐counter antioxidant that selectively targets the mitochondria and has demonstrated anxiolytic effects by reversing the molecular perturbations observed in HAB (high anxiety‐related behavior) mice.^9^, ^16^ Given the lack of effective pharmacological treatments, there is a critical need to expand our traditional understanding of anxiety disorders and develop novel treatments based on other mechanisms, including mitochondrial dysregulation, underlying anxiety disorders.

Open‐field activity (OFA) is commonly used as a proxy for measuring anxiety‐like behaviors because it utilizes the conflict situation between the innate tendencies of mice to explore a novel environment and to avoid brightly lit, open spaces.^17^ Thus, mice with low activity are models of high anxiety‐like behavior and mice with high activity serve as the counterpart model of low anxiety‐like behavior. Over 40 years ago, DeFries et al.^18^ crossed two inbred strains of mice (BALB/cJ and C57BL/6; RRID:IMSR_JAX:000651 and MGI Cat# 2159769,RRID:MGI:2159769, respectively) and bidirectionally selected for open‐field behavior. After 30 generations of selection, the High and Low Activity lines displayed 30‐fold differences in OFA.^18^ Following the original selection experiment, the lines were inbred to produce the six inbred DeFries strains: two high (H1 and H2), two low (L1 and L2) and two control lines (C1 and C2). More recently, we have^19^ confirmed that the extreme differences in OFA remain over 40 years later. In addition to extreme OFA differences, the High (H2) Activity mice exhibited low anxiety‐like behaviors and the Low (L2) Activity mice exhibited high anxiety‐like behaviors in the light–dark box (LDB) and elevated plus‐maze (EPM) paradigms, demonstrating continued support for their high and low anxiety‐like behavioral selection.^19^

A common concern about the use of the High and Low Activity mice as a model of anxiety‐related behaviors is whether the differences observed in the behavioral tests are due merely to differences in locomotion in general. To test this concern, Booher et al.^19^ also included novel object exploration, an anxiety‐like behavioral measure that does not rely heavily on locomotion. We also tested home cage voluntary wheel running in their behavioral battery. During novel object exploration, both male and female High Activity mice explore (nose poke) the novel object significantly more often than their Low Activity counterparts. This result aligns with the OFA, LDB and EPM results, where High Activity mice display low anxiety‐like behaviors and the Low Activity mice display high anxiety‐like behaviors.^19^ During the voluntary wheel running experiment, the male Low Activity mice ran nearly twice as far as the High Activity mice, while female High and Low Activity mice did not differ in wheel running.^19^ Although the male wheel running results seem counterintuitive, they suggest that the High and Low Activity mice were selected based on anxiety‐like differences and not simply on differences in generalized locomotor activity.

In addition to the recent behavioral studies, several QTL mapping studies were performed on the F2 generations of both H1XL1 and H2XL2 mice to attempt to uncover genetic loci that may harbor genes responsible for the dramatic differences in anxiety‐like behavior.^20^, ^21^, ^22^, ^23^, ^24^ Since the original selection experiment, these mice have been continuously bred at the Institute for Behavioral Genetics (IBG) at the University of Colorado Boulder. Unfortunately, the H1 and control (C1, C2) strains of the DeFries et al.^18^ study are no longer available, but the H2, L1 and L2 Activity strains of mice remain. Due to advances in technology and frozen tissue collected from the last H1 mice, we completed whole genome DNA sequencing of all four (H1, H2, L1, L2) strains, which allowed us to further refine the previously identified QTL regions to a list of 2428 genes of interest. Coupled with GeneWeaver, an integrative genomics approach, we prioritized 59 genes within the High and Low Activity QTLs as candidates for contributing to the differences between these strains.^25^

The current study builds on this recent work using hippocampal RNA sequencing as another tool to identify genes that differ between the High and Low Activity strains, using the extant H2 and L2 mice. Although the H2/L2 comparison cannot fully capture the genetic information from both pairs of replicated lines, previous QTL mapping studies of the two crosses (H1XL1 and H2XL2) suggest little to no difference between the results from the replication selection strains.^20^, ^21^, ^22^, ^23^, ^24^ In particular, tables 1–4 in Turri et al.^23^ supported this conclusion, showing similar QTL identified in the replicate F2 populations, with similar contributions to genetic variance. Furthermore, the whole‐genome sequencing study of all four strains (H1, H2, L1 and L2) revealed extremely high genome‐wide DNA similarity between the H1/H2 and L1/L2 mice.^25^

The goal of this study was to identify genes and pathways contributing to the behavioral differences between the High and Low Activity strains using RNA sequencing of naive tissue from the hippocampus of each strain. The hippocampus was selected because pathological anxiety has been demonstrated to be associated with the hippocampus, perhaps due to its key role in contextual learning/memory.^22^, ^23^, ^26^ To our knowledge, our experiment is the first RNA‐seq study performed on animals selectively bred for high and low activity/anxiety‐like behaviors.^18^, ^19^ While previous studies each provided valuable insight regarding mechanisms induced following anxiety‐like experiences, they did not focus on possible underlying differences due to genetics. The High and Low Activity mice provide an opportunity to identify baseline differences between animals with highly divergent levels of anxiety‐like responses. In addition, previous genetic mapping studies reproducibly identified QTL regions contributing to these anxiety‐like behaviors, but resolution was limited because only two parental strains (BALB/cJ and C57BL/6) were used during the original selection.^20^, ^21^, ^22^, ^23^, ^24^ Thus, by combining the results from the current hippocampal RNA‐seq study with previously identified QTL regions^21^ and whole‐genome sequencing data,^25^ we will be able to further prioritize genes that may underlie the behavioral differences in the High and Low Activity strains of mice.

## 2 MATERIALS AND METHODS

The ARRIVE Essential 10 guidelines is provided in Supplementary Table S1.

### 2.1 Animals

Sixteen total male and female High (H2) and Low (L2) Activity mice aged 70 days were used for these experiments (2 sexes × 2 levels of activity [High vs. Low] × 4 biological replicates = 16 samples). Animals were bred at the specific pathogen‐free Jennie Smoly Caruthers Biotechnology Building at the University of Colorado Boulder (AAALAC accredited institution). As soon as the mice were weaned, the mice were transferred to the specific pathogen‐free Institute for Behavioral Genetics (IBG) at the University of Colorado Boulder. Both facilities test for the following pathogens every quarter: Mouse Parvovirus (MPV), Epizootic Diarrhea of Infant Mice (EDIM), Murine Norovirus (MNV), Mouse Hepatitis Virus (MHV), Minute Virus of Mice (MVM) and Theiler's Murine Encephalomyelitis Virus (TMEV). The mice were maintained on a 12‐h light:12‐h dark cycle (lights on at 7:00 AM) and provided with ad libitum access to food (Envigo Teklad 2914 irradiated rodent diet, Harlan) and water. The mice were group housed in standard 30 cm length × 13 cm width × 17 cm height polycarbonate cages. The room temperature was maintained between 23 and 24.5 C. Using the guidelines set by the Office of Laboratory Animal Welfare, the local Institutional Animal Care and Use Committee (IACUC) approved all protocols.

### 2.2 Tissue collection and RNA extraction

At 9:00 AM, the mice were moved to the experimental room and given 1 h to acclimate. Mice were sacrificed between 10:00 AM–12:00 PM. Due to differences in coat color (High Activity = brown; Low Activity = white), experimental blinding was not possible until after brain extraction. Directly after cervical dislocation, brains were extracted and bilateral hippocampi were removed, placed in RNALater (Ambion, Foster City, California) and stored at −80°C. From this point on, and until necessary in the RNA sequencing pipeline, the experimenter was blinded to the different groups (High vs. Low Activity) and sexes. Total RNA was extracted and purified using Qiagen RNeasy Mini kits (Qiagen, Valencia, California). Quality was determined using a NanoDrop2000 spectrophotometer (ThermoFisher Scientific, Waltham MA). Ratios of absorbance (260 nm:280 nm) were shown to be excellent (>1.8).

### 2.3 Library preparation, quality control screening and high‐throughput sequencing

Twenty RNA samples (2 sexes × 2 activity levels [High vs. Low] × 5 biological replicates = 20 samples) were sent to Novogene (Sacramento, California) for quality control screening, library preparation and high‐throughput sequencing. The integrity and quantity of RNA in all samples were determined using an Agilent 2100 Bioanalyzer (RRID:SCR_019389) and all 20 samples were shown to be excellent (RIN > 7.8). Due to funding constraints, only 16 of the highest quality samples (2 sexes × 2 activity levels [High vs. Low] × 4 biological replicates = 16 samples) were included for further processing.^27^ Polyadenylated RNA was selected from total RNA using oligo (dt) beads and library construction was conducted using a NEBNext Ultra II RNA Library Prep Kit (New England Biolabs, Ipswich, MA) according to a modified protocol.^28^ According to the modified protocol, double‐stranded cDNA libraries were prepared from fragmented mRNA by reverse transcription, subsequent second strand cDNA synthesis, terminal (end) repair, A‐overhang ligation and sequencing adaptor ligation. The resulting cDNA libraries were then size‐selected (150 bp) and amplified by polymerase chain reaction (PCR). Library quality was assessed using the Agilent 2100 Bioanalyzer and qPCR was conducted to quantify the effective cDNA library concentrations. Qualified cDNA libraries were then paired‐end sequenced^28^ on the Illumina NovaSEQ6000 (RRID:SCR_020150;150 base pair paired‐end reads). Raw high‐throughput sequencing data were transformed to sequenced reads via base calling and recorded in FASTQ files provided by Novogene.^29^

### 2.4 Bioinformatic analyses

Preceding alignment, reads were de‐multiplexed and trimmed of low quality base calls and adaptors using cutadapt (RRID:SCR_011841; Version 3.1).^30^ Entire reads were eliminated if either read fragment was less than 20 bp after trimming. Additionally, quality of raw reads was assessed using FastQC (RRID:SCR_014583; Version 0.11.9).^31^ Trimmed reads were aligned via HISAT2 (RRID:SCR_015530; Version 2.2.1) to the mm10 version (Dec 2019) of the mouse genome downloaded from the UCSC Genome Bioinformatics Site (http://genome.ucsc.edu).^32^ The trimmed reads were aligned to the Ensembl mouse transcriptome (GRCm38.93) and gene‐level estimates of abundance were derived using RNA‐seq by Expectation–Maximization (RSEM; RRID:SCR_013027; Version 1.2.31).^33^ All further statistical analyses of expected gene counts were conducted in R Statistical Software (RRID:SCR_001905; Version 4.0.5). Any gene with an expected read count of zero across all samples was removed. As another form of quality control, the data was visualized by density plot, dendrogram (Supplementary Figure S1), relative log expression (RLE) plot and a principal component (PC) analysis.

**FIGURE 1:**
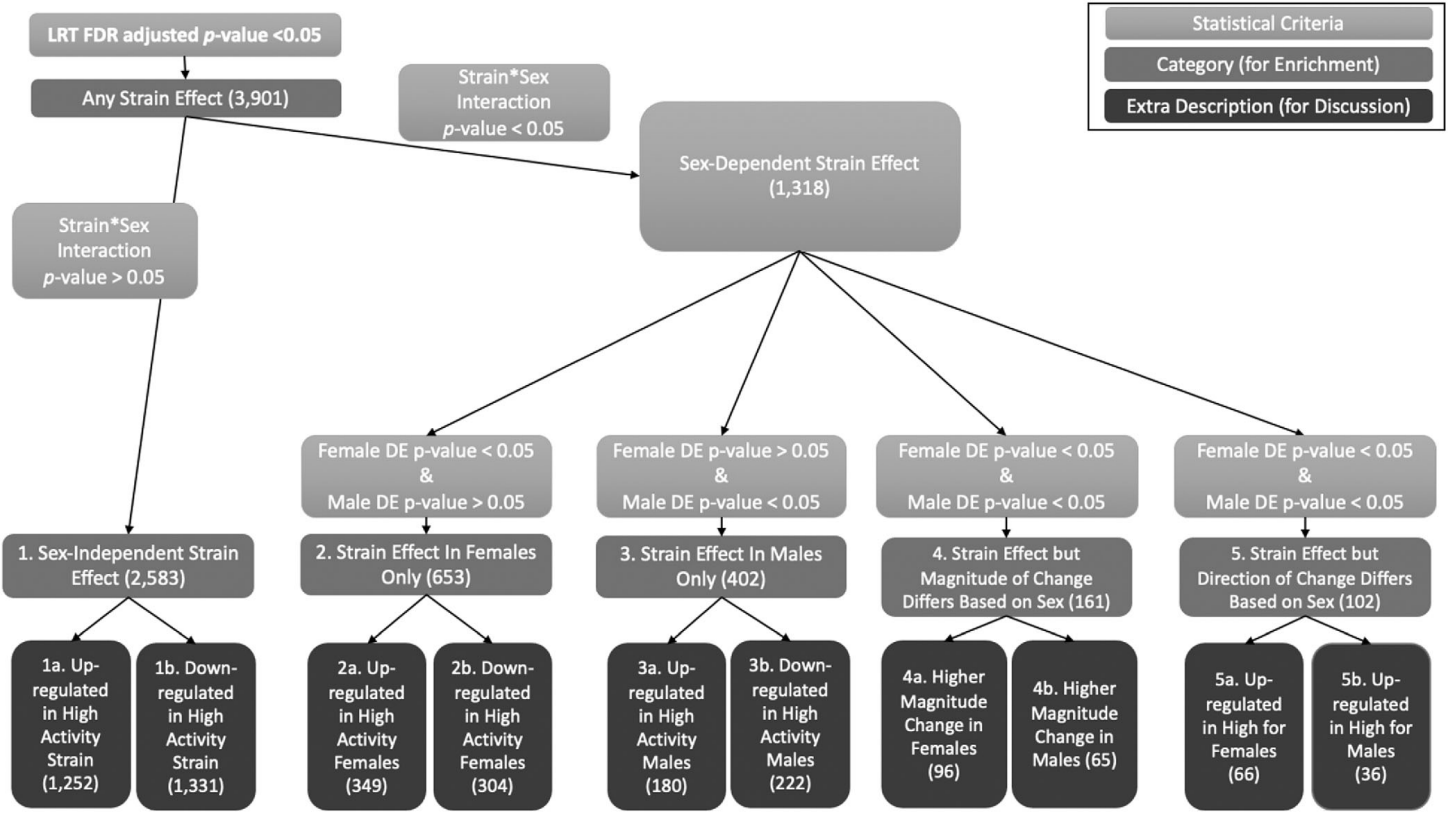
Likelihood ratio test (LRT) statistical criteria and categories (number of genes associated with each category in parentheses)

We identified strain effects (dependent or independent of sex) on gene expression by performing a likelihood ratio test (LRT) using DESeq2 (RRID:SCR_015687; Version 1.30.1),^34^ with a full model that includes strain, sex and the interaction between strain and sex as predictors of gene expression, while the reduced model only included the main effect of sex. We controlled for multiple testing by using a False Discovery Rate^35^ and candidates were those with a LRT FDR < 0.05. We further classified these candidates based on the type of strain effect they exhibited using the interaction term between strain and sex and the direction of strain effects stratified by sex (Figure 1, analysis flow chart). Those with a nominally significant strain by sex interaction (*p*‐value < 0.05) were classified as sex dependent candidates and further described based on sex stratified analyses. Those without a nominally significant strain by sex interaction (*p*‐value >0.05) are considered sex independent candidates. Please see Figure 1 for more details. Supplementary Table S2 provides summary and descriptive statistics for each gene/experimental group and comparisons between groups (e.g., median, mean, rlog, log2FoldChange, lfcSE, *p*‐value and *p*‐adjusted).

Three protein‐coding gene lists were created (hereafter referred to as “any strain effect” LRT FDR <0.05), and more specifically “sex‐independent strain effect” (LRT FDR <0.05 and strain × sex interaction *p*‐value > 0.05), and “sex‐dependent strain effect” (LRT FDR < 0.05 and strain × sex interaction *p*‐value < 0.05).

### 2.5 PANTHER gene ontology and enrichment

PANTHER (Protein Analysis Through Evolutionary Relationships; RRID:SCR_004869) identifies gene ontology (GO) terms that are statistically overrepresented by gene products within a list of candidate genes. Using the three gene lists curated from the DESeq2 analysis (any strain effect, sex‐independent strain effect and sex‐dependent strain effect), PANTHER's statistical overrepresentation test was used to determine enrichment of PANTHER pathways and GO terms. GO terms were classified as either related to biological processes or molecular functions. Using the binomial distribution, PANTHER compares a gene list to a set of reference genes. For this analysis, our reference set was constrained to the protein‐coding genes included in the mm10 version (Dec 2019) of the mouse genome downloaded from the UCSC Genome Bioinformatics Site (http://genome.ucsc.edu). Therefore, when many genes within the gene list share a common pathway or GO term, one can hypothesize that the collective function of the gene list is related to the ontology term or pathway. We considered terms/pathways with a Bonferroni adjusted *p*‐value < 0.05, and an expected fold enrichment >2.^36^ Because the statistical overrepresentation test can produce a significant result if only one gene is associated with a GO term or pathway, we implemented an additional criterion that at least five genes within the candidate gene list needed to be associated with the GO term or pathway.^36^ Finally, to reduce the redundancy of the hierarchical structure of the GO terms, we only reported the significant child terms if the parent and the child term were significant.

### 2.6 Comparison to gene lists acquired from previous studies

As mentioned above, several QTL mapping studies were performed on the F2 generations of both H1 × L1 and H2 × L2.^20^, ^21^, ^22^, ^23^, ^24^ With recent advances in technology and frozen tissue, we completed whole‐genome sequencing of all four (H1, H2, L1, L2) strains.^25^ Thus, by combining the whole‐genome sequencing results with the previously identified QTL, we were able to create a list of 2428 genes (hereafter referred to as “QTL” gene list).^21^, ^25^ Furthermore, Thomas et al.^25^ used GeneWeaver, an integrative genomics approach, to curate a list of 59 highly connected genes (hereafter referred to as “GeneWeaver” gene list). The gene list was derived from 55 mouse, rat, and human studies (all drug free) of anxiety‐related behaviors using the search terms “anxiety,” “open‐field behavior,” “open‐field,” “elevated plus‐maze,” “elevated zero‐maze,” “light–dark box,” light/dark box” and “social interaction test.” To be included in the GeneWeaver gene list, the gene had to appear in 6 or more of the 55 studies. These 59 genes are listed in Supplemental table III, and the publications they were acquired from are listed in Supplemental table IV of Thomas et al.^25^ Additionally, figure 4 of Thomas et al.^25^ depicts the networks between the 59 most highly connected genes.

### 2.7 Rank‐rank hypergeometric overlap (RRHO)

Current techniques, including those used in this study (DESeq2), compare expression profiles with a fixed differential expression threshold to summarize the results.^37^ However, these thresholds may be too stringent, causing small but concordant patterns of gene expression to be missed. While these concordant changes may be small, they could still have biological relevance and should be explored.^37^, ^38^ Thus, in addition to conventional differential expression techniques, we have also included a rank‐rank hypergeometric overlap (RRHO; RRID:SCR_014024) analysis (Version 1.0). RRHO is an agnostic, threshold‐free algorithm, which ranks two gene lists by their *p*‐value and effect size direction, while consecutively measuring the statistical significance of the number of overlapping genes.^37^, ^38^ An RRHO analysis was performed for both strain and sex. The strain analysis compares the male High versus Low Activity mice and the female High versus Low Activity mice. The sex analysis compares the male High Activity versus female High Activity mice and male Low Activity versus female Low Activity mice. The output is a four‐quadrant heat map displaying the strength of the concordant and discordant gene expression.^37^

## 3 RESULTS

### 3.1 RNA‐sequencing

We quantitatively sequenced mRNA from 16 hippocampal samples from four groups of mice (male and female High and Low Activity, four each). Hippocampal RNA‐sequencing of the High and Low Activity mice began with approximately 515 million paired‐end reads (1.03 billion read fragments). After trimming, approximately 930 million (90%) read fragments aligned to the Ensembl mouse transcriptome (GRCm38.93), including 19 autosomal chromosomes and 2 sex chromosomes. After the genes with an expected read count of zero across all samples were removed, 31,960 genes remained.

### 3.2 Differential expression analysis

A likelihood ratio test (LRT) was run via DESeq2 to examine differential gene expression. The LRT revealed 3901 protein coding Ensembl genes that were differentially expressed. Of those 3901 protein coding genes, 2583 genes were differentially expressed due to a sex‐independent strain effect (i.e., no interaction between sex and strain) and 1318 genes had a sex‐dependent difference between strains (i.e., a significant strain and sex interaction effect). Additional categories were included to further elucidate differential gene expression patterns. For simplicity, we only report the gene expression patterns in the High Activity mice; however, if a gene is reported to be downregulated in High Activity mice, that is equivalent to reporting that the gene is upregulated in Low Activity mice (and vice versa). Among those genes with a strain effect that did not differ between sexes, 1252 genes were upregulated in High Activity mice (Figure 1(1a)) and 1331 genes were downregulated in High Activity mice (Figure 1(1b)). Among those genes that showed a sex‐dependent strain effect, 349 genes were upregulated in female High Activity mice (Figure 1(2a)) and 304 genes were downregulated in female High Activity mice (Figure 1(2b)). 180 genes were upregulated in male High Activity mice (Figure 1(3a)) and 222 genes were downregulated in male High Activity mice (Figure 1(3b)). 96 genes had a higher magnitude change in female mice (Figure 1(4a)) and 65 genes had a higher magnitude change in males (Figure 1(4b)). 66 genes were upregulated in female High Activity mice but downregulated in male High Activity mice (Figure 1(5a)). Finally, 36 genes were upregulated in male High Activity mice but downregulated in female High Activity mice (Figure 1(5b)).

### 3.3 PANTHER gene ontology and enrichment

We considered terms/pathways containing at least 5 gene candidates in the term as candidates, with a Bonferroni adjusted *p*‐value < 0.05, and an expected fold enrichment >2.^36^ Here we report the GO terms and PANTHER pathways associated with the three gene lists created from the LRT via DESeq2 (Supplementary Table S3).

#### 3.3.1 Any strain effect: 3901 genes

The enrichment analysis of the any strain effect gene list indicated an enrichment of two biological process GO terms, mitochondrial ATP synthesis coupled electron transport (GO:0042775) and axonal transport (GO:0098930). 26 genes were associated with mitochondrial ATP synthesis coupled electron transport (GO:0042775), representing 3.00‐fold enrichment for the category (Bonferroni adjusted *p*‐value = 0.013). 28 genes were associated with axonal transport (GO:0098930), representing 2.76‐fold enrichment for the category (Bonferroni adjusted *p*‐value = 0.024).

The enrichment analysis of the any strain effect gene list also indicated an enrichment of one molecular function GO term, protein phosphorylated amino acid binding (GO:0045309). 29 genes were associated with protein phosphorylated amino acid binding (GO:0045309), representing 2.97‐fold enrichment for the category (Bonferroni adjusted *p*‐value = 0.0013).

#### 3.3.2 Sex‐independent strain effect: 2583 genes

The enrichment analysis of the sex‐independent strain effect gene list indicated an enrichment of two biological processes GO terms, positive regulation of neuron projection development (GO:0010976) and negative regulation of apoptotic signaling pathway (GO:2001234). 51 genes were associated with positive regulation of neuron projection development (GO:0010976), representing 2.11‐fold enrichment for the category (Bonferroni adjusted *p*‐value = 0.010). 52 genes were associated with negative regulation of apoptotic signaling pathway (GO:2001234), representing 2.03‐fold enrichment for the category (Bonferroni adjusted *p*‐value = 0.022).

The enrichment analysis of the sex‐independent strain effect gene list indicated an enrichment of one molecular function GO term, protein phosphorylated amino acid binding (GO:0045309). 22 genes were associated with protein phosphorylated amino acid binding (GO:0045309), representing 3.39‐fold enrichment for the category (Bonferroni adjusted *p*‐value = 0.0035).

#### 3.3.3 Sex‐dependent strain effect: 1318 genes

The enrichment analysis of the sex‐dependent strain effect gene list indicated an enrichment of five biological processes GO terms, mitochondrial electron transport, NADH to ubiquinone (GO:0006120), ATP synthesis coupled proton transport (GO:0015986), mitochondrial respiratory chain complex I assembly (GO:0032981), chromatin silencing (GO:0006342) and axonogenesis (GO:0007409). 9 genes were associated with mitochondrial electron transport, NADH to ubiquinone (GO:0006120), representing 8.49‐fold enrichment for the category (Bonferroni adjusted *p*‐value = 0.015). 10 genes were associated with ATP synthesis coupled proton transport (GO:0015986), representing 8.02‐fold enrichment for the category (Bonferroni adjusted *p*‐value = 0.0068). 16 genes were associated with mitochondrial respiratory chain complex I assembly (GO:0032981), representing 5.83‐fold enrichment for the category (Bonferroni adjusted *p*‐value = 0.00030). 18 genes were associated with chromatin silencing (GO:0006342), representing 4.12‐fold enrichment for the category (Bonferroni adjusted *p*‐value = 0.0068). 43 genes were associated with axonogenesis (GO:0007409), representing 2.20‐fold enrichment for the category (Bonferroni adjusted *p*‐value = 0.022).

The enrichment analysis of the sex‐dependent strain effect gene list indicated an enrichment of four molecular function GO terms, proton‐transporting ATP synthase activity, rotational mechanism (GO:0046933), NADH dehydrogenase (ubiquinone) activity (GO:0008137), electron transfer activity (GO:0009055) and structural constituent of ribosome (GO:0003735). 8 genes were associated with proton‐transporting ATP synthase activity, rotational mechanism (GO:0046933), representing 8.02‐fold enrichment for the category (Bonferroni adjusted *p*‐value = 0.027). 10 genes were associated with NADH dehydrogenase (ubiquinone) activity (GO:0008137), representing 6.67‐fold enrichment for the category (Bonferroni adjusted *p*‐value = 0.0073). 15 genes were associated with electron transfer activity (GO:0009055), representing 3.88‐fold enrichment for the category (Bonferroni adjusted *p*‐value = 0.035). 26 genes were associated with the structural constituent of ribosome (GO:0003735), representing 2.98‐fold enrichment for the category (Bonferroni adjusted *p*‐value = 0.0042).

The enrichment analysis of the sex‐dependent strain effect gene list indicated an enrichment of one PANTHER pathway, vasopressin synthesis (P04395). 6 genes were associated with vasopressin synthesis (P04395), representing 7.40‐fold enrichment for the category (Bonferroni adjusted *p*‐value = 0.031).

### 3.4 Differentially expressed oxidative phosphorylation genes

We performed enrichment analyses on the three protein coding gene lists generated with DESeq2 (any strain effect, sex‐independent strain effect and sex‐dependent effect only). With the significance criteria specified above, we created a list of 264 genes that were associated with 15 GO terms and 1 PANTHER pathway for further analyses (hereafter referred to as the “top candidates” gene list). Of the 264 top candidate genes, many of the genes (46, 17%) were associated with oxidative phosphorylation. Gene symbols, gene names, encoded protein function and differential expression are listed in Table 1.

**TABLE 1:** Glossary of gene symbols, gene names and encoded protein functions for differentially expressed oxidative phosphorylation genes

Differentially Expressed Oxidative Phosphorylation Genes					
Symbol (name)	Encoded protein function	Differential gene expression pattern	Symbol (name)	Encoded protein function	Differential gene expression pattern
**Complex I**			**Complex III**		
*Ndufa1* (NADH:ubiquinone oxidoreductase subunit A1)	Encodes the supernumerary subunit A1 of complex I	Strain effect but direction differs based on sex (upregulated in High Activity males)	*Cyc1* (Cytochrome C1)	Encodes subunit 4 of complex III	Strain effect in females only (upregulated in Low Activity females)
*Ndufa2* (NADH:ubiquinone oxidoreductase subunit A2)	Encodes the supernumerary subunit A2 of complex I	Strain effect in females only (upregulated in Low Activity females)	*Uqcc3* (Ubiquinol‐Cytochrome C Reductase Complex Assembly Factor 3)	Involved in the assembly of complex III	Strain effect independent of sex (upregulated in Low Activity mice)
*Ndufa3* (NADH:ubiquinone oxidoreductase subunit A3)	Encodes the supernumerary subunit A3 of complex I	Strain effect in males only (upregulated in High Activity mice)	*Uqcr10* (Ubiquinol‐Cytochrome C Reductase, Complex III Subunit X)	Encodes subunit X of complex III	Strain effect but direction differs based on sex (upregulated in High Activity males)
*Ndufa4* (NADH:ubiquinone oxidoreductase subunit A4)	Protein function still in question	Strain effect in females only (upregulated in Low Activity females)	*Uqcr11* (Ubiquinol‐Cytochrome C Reductase, Complex III Subunit XI)	Encodes subunit XI of complex III	Strain effect in females only (upregulated in Low Activity females)
*Ndufa6* (NADH:ubiquinone oxidoreductase subunit A6)	Encodes the supernumerary subunit A6 of complex I	Strain effect in females only (upregulated in Low Activity females)	*Uqcrfs1* (Ubiquinol‐Cytochrome C Reductase, Rieske Iron–Sulfur)	Encodes subunit 5 of complex III	Strain effect in females only (upregulated in Low Activity females)
*Ndufa7* (NADH:ubiquinone oxidoreductase subunit A7)	Encodes the supernumerary subunit A7 of complex I	Strain effect in females only (upregulated in Low Activity females)	*Uqcrh* (Ubiquinol‐Cytochrome C Reductase Hinge Protein)	Encodes subunit 6 of complex III	Strain effect in males only (upregulated in High Activity males)
*Ndufa8* (NADH:ubiquinone oxidoreductase subunit A8)	Encodes the supernumerary subunit A8 of complex I	Strain effect in females only (upregulated in Low Activity females)	*Uqcrq* (Ubiquinol‐Cytochrome C Reductase Complex III Subunit VII)	Encodes subunit VII of complex III	Strain effect in females only (upregulated in Low Activity females)
*Ndufa12* (NADH:ubiquinone oxidoreductase subunit A12)	Encodes the supernumerary subunit A12 of complex I	Strain effect but direction differs based on sex (upregulated in High Activity males)	**Cytochrome C**		
*Ndufa13* (NADH:ubiquinone oxidoreductase subunit A13)	Encodes the supernumerary subunit A13 of complex I	Strain effect but direction differs based on sex (upregulated in High Activity males)	*Cycs* (Cytochrome C, Somatic)	Encodes Cytochrome C, which accepts electrons from complex III and transfers them to complex IV	Strain effect in females only (upregulated in Low Activity females)
*Ndufaf8* (NADH:ubiquinone oxidoreductase complex assembly factor 8)	Involved in the assembly of complex I	Strain effect in females only (upregulated in Low Activity females)	**Complex IV**		
*Ndufb5* (NADH:ubiquinone oxidoreductase subunit B5)	Encodes the supernumerary subunit B5 of complex I	Strain effect in females only (upregulated in Low Activity females)	*Cox5a* (Cytochrome C Oxidase Subunit 5A)	Encodes subunit 5A of complex IV	Strain effect in females only (upregulated in Low Activity females)
*Ndufb6* (NADH:ubiquinone oxidoreductase subunit B6)	Encodes the supernumerary subunit B6 of complex I	Strain effect independent of sex (upregulated in Low Activity mice)	*Cox6a2* (Cytochrome C Oxidase Subunit 6A2)	Encodes subunit 6A polypeptide 2 of complex IV	Strain effect independent of sex (upregulated in High Activity mice)
*Ndufb7* (NADH:ubiquinone oxidoreductase subunit B7)	Encodes the supernumerary subunit B7 of complex I	Strain effect but direction differs based on sex (upregulated in High Activity males)	*Cox6c* (Cytochrome C Oxidase Subunit 6C)	Encodes subunit 6C of complex IV	Strain effect in females only (upregulated in Low Activity females)
*Ndufb9* (NADH:ubiquinone oxidoreductase subunit B9)	Encodes the supernumerary subunit B9 of complex I	Strain effect in females only (upregulated in Low Activity females)	*Cox7a2* (Cytochrome C Oxidase Subunit 7A2)	Encodes subunit 7A polypeptide 2 of complex IV	Strain effect in females only (upregulated in Low Activity females)
*Ndufc1* (NADH:ubiquinone oxidoreductase subunit C1)	Encodes the supernumerary subunit C1 of complex I	Strain effect in females only (upregulated in Low Activity females)	*Cox7c* (Cytochrome C Oxidase Subunit 7C)	Encodes subunit 7C of complex IV	Strain effect in females only (upregulated in Low Activity females)
*Ndufc2* (NADH:ubiquinone oxidoreductase subunit C2)	Encodes the supernumerary subunit C2 of complex I	Strain effect in females only (upregulated in Low Activity females)	*Cox8a* (Cytochrome C Oxidase Subunit 8A)	Encodes subunit 8A of complex IV	Strain effect in females only (upregulated in Low Activity females)
*Ndufs2* (NADH:ubiquinone oxidoreductase subunit S2)	Encodes the core subunit S2 of complex I	Strain effect but magnitude of change differs based on sex (Higher magntiude change in males)	**ATP Synthase (Complex V)**		
*Ndufs5* (NADH:ubiquinone oxidoreductase subunit S5)	Encodes the supernumerary subunit S5 of complex I	Strain effect in females only (upregulated in Low Activity females)	*Atp5c1* (ATP synthase F1 subunit gamma)	Encodes the gamma subunit of ATP synthase	Strain effect in females only (upregulated in Low Activity females)
*Ndufs6* (NADH:ubiquinone oxidoreductase subunit S6)	Encodes the supernumerary subunit S6 of complex I	Strain effect in females only (upregulated in Low Activity females)	*Atp5d* (ATP synthase F1 subunit delta)	Encodes the delta subunit of ATP synthase	Strain effect in females only (upregulated in Low Activity females)
*Ndufs8* (NADH:ubiquinone oxidoreductase subunit S8)	Encodes the core subunit S8 of complex I	Strain effect but direction differs based on sex (upregulated in High Activity males)	*Atp5e* (ATP synthase F1 subunit epsilon)	Encodes the epsilon subunit of ATP synthase	Strain effect in females only (upregulated in Low Activity females)
*Mt‐nd2* (Mitochondrially encoded NADH:ubiquinone oxidoreductase core subunit 2)	Encodes the core subunit 2 of complex I	Strain effect independent of sex (upregulated in Low Activity mice)	*Atp5f1* (ATP synthase peripheral stalk‐membrane subunit B)	Encodes the peripheral stalk‐membrane subunit B of ATP synthase	Strain effect in females only (upregulated in Low Activity females)
*Mt‐nd6* (Mitochondrially encoded NADH:ubiquinone oxidoreductase core subunit 6)	Encodes the core subunit 6 of complex I	Strain effect in males only (upregulated in High Activity mice)	*Atp5g3* (ATP synthase membrane subunit C locus 3)	Encodes the ATP synthase membrane subunit C locus 3	Strain effect but direction differs based on sex (upregulated in High Activity males)
**Ubiquinone**			*Atp5h* (ATP synthase peripheral stalk subunit D)	Encodes the peripheral stalk subunit D of ATP synthase	Strain effect in females only (upregulated in Low Activity females)
Coq7 (Coenzyme Q7, hydroxylase)	Encodes a protein necessary for ubiquinone biosynthesis	Strain effect independent of sex (upregulated in High Activity mice)	*Atp5j2* (ATP synthase membrane subunit F)	Encodes the membrane subunit F of ATP synthase	Strain effect but magnitude of change differs based on sex (Higher magnitude change in females)
Coq9 (Coenzyme Q9)	Encodes a protein necessary for ubiquinone biosynthesis	Strain effect in females only (upregulated in Low Activity females)	*Atp5k* (ATP synthase subunit E)	Encodes the subunit E of ATP synthase	Strain effect in males only (upregulated in High Activity males)

*Note*: Encoded protein functions were excerpted from GeneCards, Mouse Genome Informatics and Alliance of Genome Resources.

#### 3.4.1 NADH dehydrogenase (complex 1)

22 genes were associated with NADH dehydrogenase (complex) I of the electron transport chain. Of those 22 genes, 19 were upregulated in Low Activity females (*Ndufa1*, *Ndufa2*, *Ndufa4*, *Ndufa6*, *Ndufa7*, *Ndufa8*, *Ndufa12*, *Ndufa13*, *Ndufaf8*, *Ndufb5*, *Ndufb6*, *Ndufb7*, *Ndufb9*, *Ndufc1*, *Ndufc2*, *Ndufs5*, *Ndufs6*, *Ndufs8* and *Mt‐nd2*), 8 were upregulated in High Activity males (*Ndufa1*, *Ndufa3*, *Ndufa12*, *Ndufa13*, *Ndufb7*, *Ndufs2*, *Ndufs8* and *Mt‐nd6*), 2 were upregulated in Low Activity males (*Ndufb6* and *Mt‐n2*), and 1 was upregulated in High Activity females (*Ndufs2*). Several genes were significantly upregulated independent of sex, which is why the number of listed genes is more than 22.

#### 3.4.2 Ubiquinone biosynthesis

Two genes were associated with ubiquinone biosynthesis. Ubiquinone is an electron carrier from both complexes I and II to complex III. Of those two genes, *Coq7* was upregulated in both male and female High Activity mice and *Coq9* was upregulated in female Low Activity mice.

#### 3.4.3 Ubiquinol‐cytochrome c reductase (complex III)

Seven genes were associated with ubiquinol‐cytochrome c reductase (complex III) of the electron transport chain. Of these seven genes, six were upregulated in Low Activity females (*Cyc1*, *Uqcc3*, *Uqcr10*, *Uqcr11*, *Uqcrfs1* and *Uqcrq*), two were upregulated in High Activity males (*Uqcr10 and Uqcrh*), one was upregulated in Low Activity males (*Uqcc3*). In many cases, genes were significantly upregulated independent of sex, which is why the number of listed genes is more than seven.

#### 3.4.4 Cytochrome c

One gene was associated with cytochrome c. Cytochrome c transports electrons from complex III to complex IV. *Cycs* was upregulated in female Low Activity mice.

#### 3.4.5 Cytochrome c oxidase (complex IV)

Six genes were associated with cytochrome c oxidase (complex IV). Of these six genes, five were upregulated in Low Activity females (*Cox5a*, *Cox6c*, *Cox7a2*, *Cox7c* and *Cox8a*), one was upregulated in High Activity females (*Cox6a2*), and one was upregulated in High Activity males (*Cox6a2*). In one case, a gene was significantly upregulated independent of sex, which is why the number of listed genes is more than six.

#### 3.4.6 ATP synthase (complex V)

Eight genes were associated with ATP synthase (complex V). Of these eight genes, seven were upregulated in Low Activity females (*Atp5c1*, *Atp5d*, *Atp5e*, *Atp5f1*, *Atp5g3*, *Atp5h* and *Atp5j2*), two were upregulated in High Activity males (*Atp5g3 and Atp5k*), and one was upregulated in Low Activity males (*Atp5j2*). The number of genes listed is more than eight because several genes were significantly upregulated independent of sex.

### 3.5 Overlap between the top candidates, QTL and GeneWeaver gene lists

**FIGURE 2:**
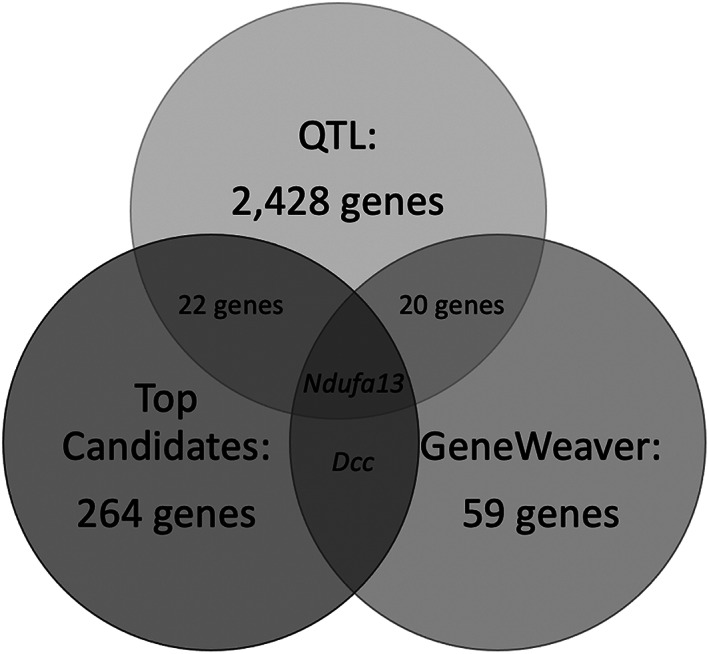
The top candidate genes (left) were compared to the QTL (top—22 overlapping genes) and GeneWeaver (right—one overlapping gene (*Dcc*)) gene lists. Additionally, all three gene lists were compared, revealing one common gene (*Ndufa13*). See Table 2 for the list of 24 genes

In order to further narrow down the priority genes, we manually compared the top candidates, QTL and GeneWeaver gene lists (Figure 2). Gene symbols, gene names, encoded protein function and differential gene expression patterns can be seen in Table 2.

**TABLE 2:** Glossary of gene symbols, gene names, encoded protein functions and differential gene expression patterns for the top candidate genes

Overlapping gene lists		
Symbol (name)	Encoded protein function	Differential gene expression pattern
**Top candidates and QTL gene list overlap**		
*Ap3s2* (Adaptor‐related protein complex 3, sigma 2 subunit)	Involved in anterograde synaptic vesicle transport. Predicted to localize to AP‐3 adaptor complex and intracellular membrane‐bounded organelle.	Strain effect independent of sex (upregulated in Low Activity)
*Arrb1* (Arrestin, beta 1)	Exhibits cysteine‐type endopeptidase inhibitor activity involved in apoptotic process and mitogen‐activated protein kinase kinase binding activity. Localizes to cytosol and pseudopodium.	Strain effect independent of sex (upregulated in High Activity)
*Coq7* (Coenzyme Q7, hydroxylase)	Predicted to have 2‐octoprenyl‐3‐methyl‐6‐methoxy‐1,4‐benzoquinone hydroxylase activity and chromatin binding activity. Localizes to mitochondrion.	Strain effect independent of sex (upregulated in High Activity)
*Crtc1* (CREB regulated transcription coactivator 1)	Predicted to have cAMP response element binding protein binding activity. Localizes to cytoplasm and nucleus. Predicted to colocalize with dendrite and synapse.	Strain effect independent of sex (upregulated in Low Activity)
*Dlg2* (Discs large MAGUK scaffold protein 2)	A structural constituent of postsynaptic density. Localizes to several cellular components, including glutamatergic synapse; juxtaparanode region of axon; and synaptic vesicle membrane. Predicted to colocalize with ionotropic glutamate receptor complex and voltage‐gated potassium channel complex.	Strain effect independent of sex (upregulated in High Activity)
*Enah* (ENAH Actin regulator)	Exhibits SH3 domain binding activity and profilin binding activity. Localizes to several cellular components, including focal adhesion; lamellipodium; and stress fiber.	Strain effect in females only (upregulated in High Activity)
*Gprc5b* (G protein‐coupled receptor, family C, group 5, member B)	Exhibits protein kinase binding activity. Localizes to cell surface; membrane raft; and plasma membrane.	Strain effect independent of sex (upregulated in High Activity)
*Lrp8* (Low density lipoprotein receptor‐related protein 8, apolipoprotein e receptor)	Exhibits several functions, including amyloid‐beta binding activity; apolipoprotein binding activity; and reelin receptor activity. Localizes to cell surface and extracellular space.	Strain effect independent of sex (upregulated in Low Activity)
*Ndufb7* (NADH:ubiquinone oxidoreductase subunit B7)	Predicted to have NADH dehydrogenase (ubiquinone) activity. Predicted to be involved in mitochondrial respiratory chain complex I assembly. Localizes to mitochondrial inner membrane.	Strain effect but direction differs based on sex (upregulted in High Activity males and Low Activity females)
*Ndufc2* (NADH:ubiquinone oxidoreductase subunit C2)	Predicted to have NADH dehydrogenase (ubiquinone) activity. Predicted to be involved in several processes, including mitochondrial respiratory chain complex I assembly; negative regulation of NIK/NF‐kappaB signaling; and positive regulation of mitochondrial membrane potential. Localizes to mitochondrial inner membrane.	Strain effect in females only (upregulated in High Activity)
*Ndufs2* (NADH:ubiquinone oxidoreductase core subunit S2)	Predicted to contribute to NADH dehydrogenase activity and to be involved in mitochondrial electron transport, NADH to ubiquinone and response to oxidative stress. Localizes to mitochondrion.	Strain effect but magnitude of change differs based on sex (upregulated in High Activity)
*Nol3* (Nucleolar protein 3)	Exhibits death effector domain binding activity and death receptor binding activity. Localizes to mitochondrion and sarcoplasm.	Strain effect independent of sex (upregulated in Low Activity)
*Ntrk3* (Neurotrophic tyrosine kinase, receptor, type 3)	Predicted to have several functions, including GPI‐linked ephrin receptor activity; neurotrophin receptor activity; and p53 binding activity. Localizes to cytoplasm.	Strain effect independent of sex (upregulated in High Activity)
*Picalm* (Phosphatidylinositol binding clathrin assembly protein)	Predicted to have several functions, including SH3 domain binding activity; clathrin heavy chain binding activity; and phosphatidylinositol binding activity. Localizes to clathrin‐coated pit and vesicle. Colocalizes with perinuclear region of cytoplasm.	Strain effect in females only (upregulated in High Activity)
*Rgma* (Repulsive guidance molecule family member A)	Exhibits coreceptor activity and signaling receptor binding activity. Localizes to cell surface.	Strain effect independent of sex (upregulated in High Activity)
*Rit2* (Ras‐like without CAAX 2)	Exhibits several functions, including GTP binding activity; GTPase activity; and calmodulin binding activity. Localizes to several cellular components, including cell body; dendritic tree; and nucleus.	Strain effect independent of sex (upregulated in Low Activity)
*Rpl27a* (Ribosomal protein L27A)	A structural constituent of ribosome. Involved in translation. Localizes to cytosolic ribosome.	Strain effect in females only (upregulated in Low Activity)
*Spcs2* (Signal peptidase complex subunit 2 homolog (S. cerevisiae))	Predicted to contribute to peptidase activity. Predicted to be involved in protein targeting to ER and signal peptide processing. Predicted to localize to signal peptidase complex.	Strain effect but magnitude of change differs based on sex (upregulated in High Activity)
*Tmem161a* (Transmembrane protein 161A)	Predicted to be involved in several processes, including cellular response to UV; regulation of response to DNA damage stimulus; and response to retinoic acid. Predicted to localize to integral component of membrane.	Strain effect independent of sex (upregulated in High Activity)
*Trim32* (Tripartite motif‐containing 32)	Exhibits several functions, including myosin binding activity; translation initiation factor binding activity; and ubiquitin protein ligase activity. Localizes to nucleus and striated muscle myosin thick filament.	Strain effect independent of sex (upregulated in Low Activity)
*Txndc12* (Thioredoxin domain containing 12 (endoplasmic reticulum))	Predicted to have peptide disulfide oxidoreductase activity. Predicted to be involved in negative regulation of endoplasmic reticulum stress‐induced intrinsic apoptotic signaling pathway. Localizes to endoplasmic reticulum.	Strain effect independent of sex (upregulated in High Activity)
*Vcl* (Vinculin)	Predicted to have alpha‐catenin binding activity; dystroglycan binding activity; and enzyme binding activity. Localizes to several cellular components, including costamere; fascia adherens; and focal adhesion. Predicted to colocalize with Actin filament and stress fiber.	Strain effect in females only (upregulated in High Activity)
**Top candidates and GeneWeaver gene list overlap**		
*Dcc* (Deleted in colorectal carcinoma)	Predicted to have identical protein binding activity; netrin receptor activity; and transcription coactivator activity. Localizes to Schaffer collateral ‐ CA1 synapse; axon; and integral component of postsynaptic density membrane.	Strain effect (upregulated in High Activity)
**Top candidates, QTL and GeneWeaver gene list overlap**		
*Ndufa13* (NADH:ubiquinone oxidoreductase subunit A13)	Predicted to have NADH dehydrogenase (ubiquinone) activity. Involved in extrinsic apoptotic signaling pathway. Localizes to mitochondrion and nucleus.	Strain effect but direction differs based on sex (upregulated in High Activity males and Low Activity females)

*Note*: Encoded protein functions were obtained from Mouse Genome Informatics and alliance of genome resources.

#### 3.5.1 Top candidates and QTL gene list overlap

22 genes were present in both the top candidates and QTL gene lists. 14 genes were upregulated in High Activity females (*Arrb1*, *Coq7*, *Dlg2*, *Enah*, *Gprc5b*, *Ndufc2*, *Ndufs2*, *Ntrk3*, *Picalm*, *Rgma*, *Spcs2*, *Tmem161a*, *Txndc12* and *Vcl*), 11 genes were upregulated in High activity males (*Arrb1*, *Coq7*, *Dlg2*, *Gprc5b*, *Ndufb7*, *Ndufs2*, *Ntrk3*, *Rgma*, *Spcs2*, *Tmem161a* and *Txndc12*), eight genes were upregulated in Low Activity females (*Ap3s2*, *Crtc1*, *Lrp8*, *Ndufb7*, *Nol3*, *Rit2*, *Rpl27a* and *Trim32*), and six were upregulated in Low Activity males (*Ap3s2*, *Crtc1*, *Lrp8*, *Nol3*, *Rit2* and *Trim32*). Several genes were significantly upregulated independent of sex, which is why the number of listed genes is more than 22.

#### 3.5.2 Top candidates and GeneWeaver gene list overlap

One gene was present in the top candidates and GeneWeaver gene lists. *Dcc* was upregulated in both male and female High Activity mice.

#### 3.5.3 Top candidates, QTL and GeneWeaver gene list overlap

One gene was present in the top candidates, QTL and GeneWeaver gene lists. *Ndufa13* was upregulated in male High Activity mice and female Low Activity mice.

### 3.6 Rank‐rank hypergeometric overlap (RRHO)

Approximately 29,000 genes were used for our RRHO analyses. In addition, we used the Stratified method extensively explained in Cahill et al.^38^

#### 3.6.1 RRHO strain results

**FIGURE 3:**
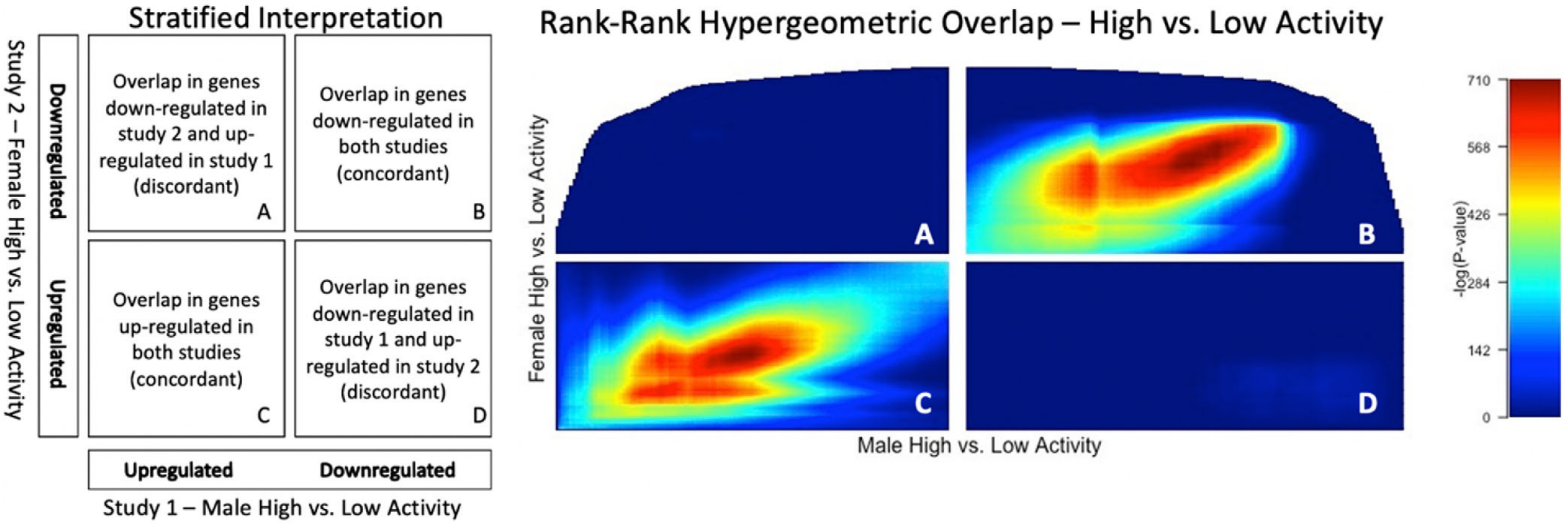
The left figure describes the differential gene expression that is represented in the RRHO heat map output, high versus low activity mice. Each pixel in the stratified heat maps below represent a −log10(*p*‐value) from the hypergeometric distribution

An RRHO strain analysis revealed concordant gene expression. The same genes are either downregulated (Figure 3B) or upregulated (Figure 3C) for each activity level in males and in females. In other words, quadrant B demonstrates that if a gene is downregulated in male High Activity versus Low Activity mice, the same gene will show the identical pattern in the female High Activity versus Low Activity mice. Quadrant C displays the opposite, if a gene is upregulated in male High Activity versus Low Activity mice, the same gene will show the identical pattern in the female High Activity versus Low Activity mice.

#### 3.6.2 RRHO sex results

**FIGURE 4:**
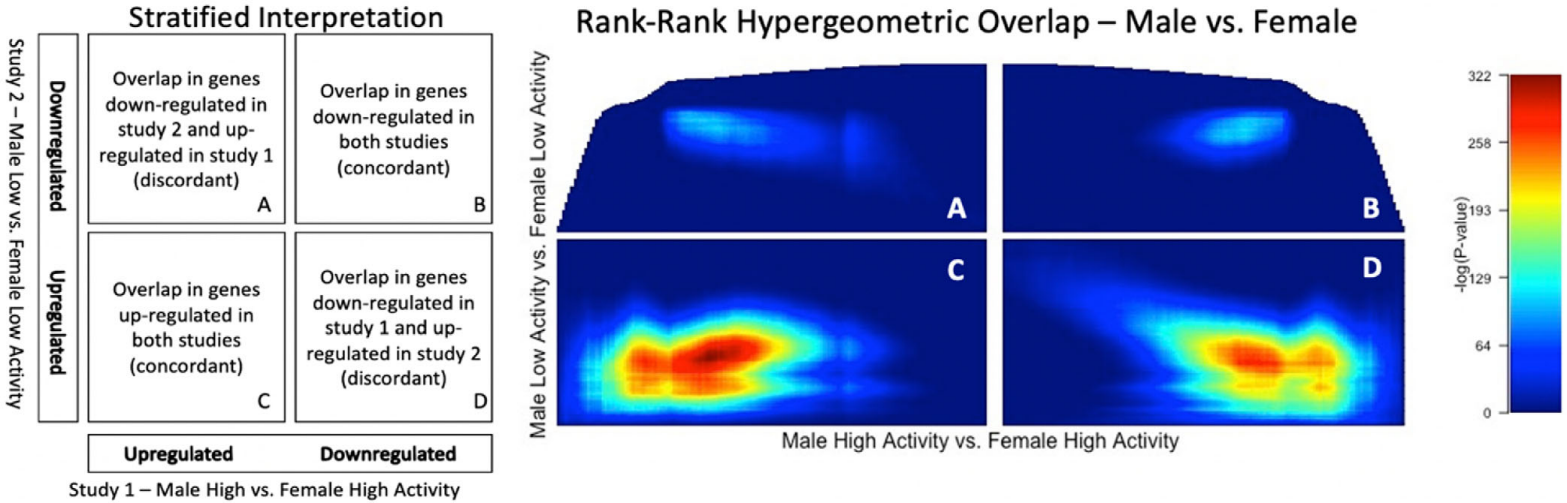
The left figure describes the differential gene expression that is represented in the RRHO heat map output, male versus female High and Low Activity mice. Each pixel in the stratified heat maps below represent a −log10 (*p*‐value) from the hypergeometric distribution

The RRHO sex analysis results are a bit more complex, revealing both concordant (Figure 4B,C) and discordant (Figure 4A,D) gene expression. Quadrant C reveals that many genes are upregulated for male versus female mice, regardless of activity strain. In other words, if a gene is upregulated in male High Activity versus female High Activity mice, the same gene will show the identical pattern for male Low Activity versus female Low Activity mice. Quadrant B again demonstrates concordant gene expression, but it is more understated than in quadrant C. More specifically, if a gene is downregulated in male High Activity versus female High Activity mice, the same gene will show the identical pattern in male Low Activity versus female Low Activity mice.

Quadrant D indicates that there are many genes downregulated in male High Activity versus female High Activity mice that are upregulated in male Low Activity versus female Low Activity mice. Quadrant A demonstrates that this pattern does not exist nearly as strong for female mice.

## 4 DISCUSSION

Hippocampal RNA sequencing demonstrated both sex‐independent and sex‐dependent differences between male and female High and Low Activity mice. A likelihood ratio test (LRT) via DESeq2 revealed 3901 significant differentially expressed protein‐coding genes. Of those genes, 2583 had a sex‐independent effect and 1318 had a sex‐dependent effect (Figure 1). The functional enrichment analysis revealed that genes related to mitochondrial ATP synthesis coupled electron transport (GO:0042775) were involved in modulating differences due to any strain effects. In addition, several other mitochondrial‐related ontologies were identified for the sex‐dependent strain effects: mitochondrial electron transport, NADH to ubiquinone (GO:0006120), ATP synthesis coupled proton transport (GO:0015986), mitochondrial respiratory chain complex I assembly (GO:0032981). We then focused on the 264 protein‐coding genes that were both differentially expressed and were associated with one of the enriched GO terms or the enriched PANTHER pathway. 46 of the top candidates genes were associated with oxidative phosphorylation, and 39 (3 sex‐independent and 36 sex‐dependent) of the 46 genes were upregulated in female Low Activity mice (Table 1). Additionally, using the previous QTL and GeneWeaver gene lists generated by Thomas et al.,^25^ 22 genes were present in both the top candidates and QTL gene lists, 1 gene (*Dcc*) was present in both the top candidates and GeneWeaver gene lists (upregulated in male and female High Activity mice), and 1 gene (*Ndufa13*) was present in all three lists (upregulated in male High Activity and female Low Activity mice) (Figure 2 and Table 2). In summary, the High and Low Activity mice display highly divergent levels of anxiety‐like responses,^19^ and this hippocampal RNA‐seq study provided an opportunity to identify baseline differences that may underlie the high and low anxiety‐like phenotypes.

This study focuses on the role of the hippocampus, because pathological anxiety has been demonstrated to be associated with the hippocampus, perhaps due to its key role in contextual learning/memory.^26^, ^39^, ^40^ Differential gene expression studies using CSDS models have revealed important changes in mitochondrial‐related gene pathways in the hippocampus.^41^, ^42^ In addition, by using the forced swimming test (FST) or tail suspension test (TST), Zhang et al.^43^ induced anxiety‐related behaviors and the altered behaviors were associated with extensive transcriptional changes in the hippocampus. More specifically, the hippocampal transcriptional alterations in the TST mice were associated with pyruvate metabolism and oxidative phosphorylation, suggesting mitochondrial modifications.^43^ Taken together, the above studies demonstrate that inducing anxiety‐like behaviors via stress or trauma results in transcriptional alterations of the hippocampus associated with mitochondrial alterations.^26^, ^39^, ^40^, ^41^, ^42^, ^43^

As presented in Table 1, the vast number of differentially expressed genes involved in oxidative phosphorylation warrants further discussion regarding the implications of this molecular function on anxiety‐like behaviors. Oxidative phosphorylation is the final step of cellular respiration, which occurs in the inner mitochondrial membrane and utilizes a series of oxidation–reduction reactions to transfer electrons from NADH and FADH2 across the electron transport chain (ETC) to generate approximately 34 ATP molecules.^44^ Although oxidative phosphorylation generates the majority of ATP produced by cellular respiration, it is also a major producer of reactive oxygen species (ROS), mostly through premature electron leak, and thus is largely accountable for oxidative stress.^9^, ^45^ While ROS are a natural byproduct of cellular respiration, proper function requires a delicate balance between pro‐oxidant and antioxidant systems.^46^ When this system is perturbed, both physiological disease and psychiatric disorders can occur.^47^, ^48^, ^49^, ^50^, ^51^, ^52^ Given these points, it seems plausible that ROS may be partially responsible for the dramatic differences in anxiety‐like behaviors observed in the High and Low Activity mice.^19^

Overproduction of ROS leads to mitochondrial damage and dysfunction, including mutations of mitochondrial DNA, damage to the mitochondrial respiratory chain and mitochondrial membrane permeability.^53^ While a substantial amount of evidence has linked mitochondrial dysfunction and various psychiatric disorders,^47^, ^48^, ^49^, ^51^ the effect of mitochondrial dysfunction on anxiety disorders has not been extensively addressed.^9^ Several hypotheses have been proposed to link mitochondrial etiology and neuropsychiatric disorders.^9^, ^54^, ^55^ First, through cellular respiration, our brain consumes about 20% of our oxygen and about 25% of our glucose, while only being responsible for about 3% of our body mass. Thus, even subtle mitochondrial alterations would have a substantial negative impact on brain functions, increasing an individual's vulnerability to neuropsychiatric disorders.^9^, ^54^ Second, stress can trigger or exacerbate neuropsychiatric disorders and these disorders cause costly metabolic neural adaptations.^56^ Therefore, “a person with suboptimal mitochondrial function would be particularly vulnerable to stress‐associated depletion of the brain's energy resources, and, hence the development of psychopathologies.”^9^ Other hypotheses involve glucocorticoids,^57^ which are produced and metabolized in the mitochondria, catecholamines, which are catabolized by the mitochondria, and the critical role the mitochondria plays in hormonal stress responses.^9^, ^58^ It is unclear whether mitochondrial dysfunction causes neuropsychiatric disorders or neuropsychiatric disorders cause mitochondrial dysfunction, it is likely a combination of both that depends on the individual as well as the particular disorder.

As previously mentioned, the 264 top candidates gene list identified through the RNA sequencing analysis included 46 genes associated with oxidative phosphorylation. We separated the 46 genes into six categories all associated with oxidative phosphorylation: Complex I, ubiquinone, Complex III, cytochrome‐c, Complex IV and ATP synthase (Complex V) (Table 1). While a handful of genes were upregulated in the High Activity (14 for males and 3 for females) and male Low Activity mice (4), a striking 39 out of 46 oxidative phosphorylation genes were upregulated in female high anxiety, Low Activity mice. More specifically, 17 genes each encode a subunit of Complex I, one gene was associated with Complex I assembly (*Ndufaf8*), and one the function of one gene is still questioned (*Ndufa4*). One gene (*Coq9*) encodes a protein necessary for ubiquinone biosynthesis. Five genes each encode a subunit of Complex III, and one gene is involved in Complex III assembly (*Uqcc3*). One gene encodes cytochrome‐c (*Cycs*), five genes each encode a subunit of Complex IV, and seven genes each encode a subunit of ATP synthase (Complex V). Interestingly, only four (three sex‐indenpendent and one sex‐dependent) of the 46 genes associated with oxidative phosphorylation were upregulated in male high anxiety, Low Activity mice (two associated with Complex I, one with Complexes III and V). Thus, in addition to the strain differences observed for oxidative phosphorylation genes, this result suggests there may be a strong sex effect associated with mitochondrial dysfunction and anxiety‐like behaviors.

In addition to the 46 genes associated with oxidative phosphorylation, five of these genes also appeared in our overlapping gene lists (any of the 264 top candidate genes that overlap with the QTL and/or GeneWeaver gene lists), providing more evidence for their relevance in the different anxiety‐like behaviors observed in the High and Low Activity mice. *Ndufb7*, *Ndufc2*, *Ndufs2* and *Coq7* appeared on both the top candidates and the QTL gene list, and *Ndufa13* appeared on all three gene lists.^21^, ^25^ The four “*Nduf*” genes each encode a subunit of Complex I and *Coq7* encodes a protein necessary for ubiquinone biosynthesis, which is an electron carrier from Complexes I and II to Complex III. Interestingly, of the 46 genes associated with oxidative phosphorylation, zero were associated with Complex II, which is the only complex of the ETC that does not pump protons into the intermembrane space of the mitochondria. Collectively, our results suggest that differences in genes involved in Complexes I, III, IV and V, as well as the electron carriers, ubiquinone and cytochrome C may influence anxiety‐like behaviors and that these differences may be sex‐dependent.

Similar to the DeFries et al.^18^ mice used in our study, Krömer et al.^59^ used over 25 liters of the outbred Swiss CD1 strain as a starting point for selective and bidirectional breeding for anxiety‐related behavior on the EPM, eventually creating the inbred HAB‐M (high anxiety behavior‐EPM), LAB‐M (low anxiety behavior‐EPM) and NAB‐M (normal anxiety behavior‐EPM) mouse lines. Proteomic and microarray analysis revealed that glyoxalase‐I (Glx1), an enzyme involved in glycolysis, was consistently upregulated in LAB‐M mice in several brain regions.^59^ Glycolysis is the first step of cellular respiration and therefore closely linked to mitochondrial energy production.^9^, ^59^ In two follow up studies, male only HAB, LAB and NAB were metabolically labeled with the stable 15N isotope, via a 15N‐labeled diet, for further proteomic and metabolomic profiling.^60^, ^61^ Both studies reported cingulate cortex protein and metabolite alterations in energy metabolism, mitochondrial import and transport, and oxidative stress. More specifically, seven of the 10 enzymes catalyzing glycolysis pathway reactions had increased expression in LAB mice, with the largest change observed in Glx1. Increased protein expression of proteins participating in mitochondrial import and transport were observed in HAB mice and there was evidence for increased antioxidant activity in LAB mice.^60^, ^61^ Furthermore, in silico analysis confirmed the increased antioxidant activity in LAB mice and reports an increased fold change in HAB mice for oxidative phosphorylation and metabolic processes.^60^ In a final study using male HAB mice, Nussbaumer et al.^16^ treated HAB, C57BL/JN and DBA/2J mice with MitoQ, an over the counter antioxidant that selectively targets the mitochondria, for 10 weeks. The HAB mice treated with MitoQ exhibit decreased anxiety‐like behaviors in the LDB and OFA; however, the C57BL/JN and DBA/2J were not affected by MitoQ. This suggests that pathological levels of anxiety may be necessary for MitoQ to be effective.^16^ In summary, the HAB, NAB and LAB mice selected for their EPM behaviors provide continued support for the mitochondrial role in anxiety‐like behaviors.^16^, ^59^, ^60^

In another selective and bidirectional study, anticipatory anxiety behavior during cage changes was used to create inbred AX (anxious) and NAX (non‐anxious) lines of mice.^62^ These male mice performed as expected in EPM, LDB and OFA (AX mice exhibited increased anxiety‐like behaviors and vice versa). A significant change in 82 proteins was revealed during this study, 34 of which had been previously identified in other anxiety, depression or repeated psychosocial stress studies. Importantly, significant changes in oxidative stress proteins (GLRX3, GSTM1, PRDX6, QDPR and SIRT2) were associated with male AX mice.^62^

The mitochondria is a major source of ROS, thus Olsen et al.^63^ created mice that overexpress mitochondrial catalase (MCAT). MCAT has been shown to protect cells from oxidative injury.^64^ Both male and female MCAT mice exhibited reduced measures of anxiety in the elevated zero maze. Interestingly, oxidative stress did not significantly differ between groups, suggesting the differences observed in this study may be due to altered redox signaling.^63^ Misiewicz et al.^65^ used a chronic social defeat stress (CSDS) paradigm in male C57BL/6NCrl and DBA/2NCrl, two strains known to differ in their susceptibility to stress. Integrative gene set enrichment analysis revealed differential expression of mitochondrial‐related genes in the bed nucleus of the stria terminalis (BNST) and blood of stressed mice. Interestingly, this study found a strain dependent effect, where reduced expression of mitochondrial‐related genes were observed in DBA/2NCrl stress‐susceptible mice but increased expression of these genes in C57BL/6NCrl stress‐susceptible mice.^65^ In addition, of the mitochondrial‐related genes reported in Misiewicz et al.,^65^ many also appeared in our top candidates gene list, including five genes (*Ndufa2*, *Ndufa6*, *Ndufc1*, *Ndufc2* and *Ndufs6*) that encode Complex I subunits, one gene (*Uqcrh*) that encodes a Complex III subunit, one gene (*Cycs*) that encodes cytochrome‐c, three genes (*Cox5a*, *Cox7a2* and *Cox7c*) that encode Complex IV subunits, and three genes (*Atp5c1*, *Atp5d* and *Atp5f1*) that encode Complex V (ATP synthase) subunits. Taken together, the HAB/NAB/LAB, AX/NAX, MCAT and multiple C57/DBA strains of mice were utilized in several different paradigms, but all provide strong support that the mitochondria are involved in anxiety‐like behaviors and offers potential therapeutic targets.

In addition to the oxidative phosphorylation results presented above, we manually compared our top candidates gene list with our previously published QTL and GeneWeaver gene lists (Table 2).^25^ Niculescu et al.^66^ reported that decreased *ARRB1* expression in the prefrontal cortex (PFC) was among the top 12 list of candidate biomarker genes involved with suicidality. While our results cannot be translated directly to humans, it is interesting that *Arrb1* upregulation was observed in both male and female low anxiety, High Activity mice. In addition, the hippocampus is a major target of all‐trans retinoic acid (ATRA), which has been reported to induce anxiety‐like behaviors. Qin et al.^67^ injected young mice with 10 mg/kg of ATRA and this resulted in a significant decrease in *Dlg2* mRNA. *Dlg2* mRNA levels were positively correlated with center duration and total distance traveled in OFA, suggesting lower anxiety‐like behaviors. In agreement with our results, *Dlg2* was upregulated in low anxiety, High Activity mice. Furthermore, Fox et al.^68^, ^69^ demonstrated an inverse association between *Ntkr3* and anxious‐temperament (AT) in young rhesus monkeys. Correspondingly, this gene was found to be upregulated in both male and female low anxiety, High Activity mice. There have been mixed results reported for *Trim32*. First, TRIM32 was strongly associated with anxiety disorders based on a study of copy number variants.^70^ Ruan et al.^71^ demonstrated that TRIM32 KO mice exhibit decreased anxiety‐like behaviors under normal and moderately stressful conditions (elevated‐zero maze and OFA). However, Hillje et al.^72^ reported no difference in EPM or OFA for TRIM32 KO mice. Conversely, *Trim32* was found to be upregulated for both male and female high anxiety, Low Activity mice. Finally, *Dcc* was the only gene to appear on the top candidates and GeneWeaver gene lists, and it was found to be upregulated in low anxiety, High Activity mice. While very few studies mentioned this gene, Ward et al.^73^ conducted a genome‐wide association study (GWAS) and reported that *Dcc* has a significant role in mood stability and Misiewicz et al.^65^ reported *Dcc* as a mitochondrial‐related gene with reduced expression in DBA/2NCrl stress‐susceptible mice but increased expression in C57BL/6NCrl stress‐susceptible mice. Given the above points, the 24 genes that have appeared on multiple gene lists curated by our lab, especially those that appear in other studies, likely modulate anxiety‐like behaviors and warrant further exploration.

In our final analysis, we included RRHO because it uses an agnostic and threshold‐free algorithm that may detect small patterns of gene expression of potential biological relevance.^37^, ^38^ The first RRHO analysis examined differential gene expression between the High and Low Activity strains. Figure 3 reveals concordant gene expression between the two strains, suggesting that sex‐independent transcriptional differences largely underlie the differences in activity and anxiety‐like behaviors (Figure 3B,C). This result is confirmed by both our DESeq2 analysis and dendrogram. DESeq2 demonstrated that 2583 of the 3901 differentially expressed genes have a sex‐independent effect (Figure 1(1a,1b)), and the dendrogram depicts clustering by strain, not sex (Figure S1). The second RRHO analysis examined differential gene expression between sexes. Figure 4 displays both concordant and discordant gene expression. Here, the concordant gene expression is a signature of sexual dimorphism independent of activity phenotype (Figure 4B,C). This was also confirmed by our DESeq2 analysis because 653 of the 3901 differentially expressed genes had a female only strain effect (Figures 1 and 2) and 402 of the 3901 differentially expressed genes had a male only strain effect (Figures 1 and 3). The discordant gene expression demonstrates that many genes are down‐regulated in male High Activity mice but up‐regulated in male Low Activity mice (Figure 4D). However, this pattern does not exist nearly as strongly for females (Figure 4A). One interpretation is that the male activity phenotype is the result of more divergent transcriptional differences than the female activity phenotype. Finally, these results confirm our DESeq2 analysis because 1318 of the 3901 differentially expressed genes have a sex‐dependent effect (Figures 1, 2, 3, 4).

While there are many strengths to this model, the current RNA‐seq study has a couple of limitations. First, because only two inbred strains were used to create the High and Low Activity mice, the genetic diversity was limited to these two strains, so findings from these strains may not generalize to other strains or outbred populations of mice. Furthermore, our experimental design was limited to only two strains of mice, which is not ideal for drawing direct correlations between gene expression patterns and behavior. There are likely to be gene expression differences between the High and Low Activity strains that emerged due to chance and not due to the phenotype, so our gene lists should be interpreted with caution. Likewise, the mapping resolution possible in these strains is suboptimal for identifying specific genes within the previously identified QTLs.^21^, ^22^, ^23^, ^24^, ^74^, ^75^ However, by combining the hippocampal RNA‐seq results with the whole‐genome sequencing (H1, H2, L1 and L2),^39^ previously identified QTL regions,^21^ and integrative functional genomic analyses, we were able to prioritize a narrow list of genes.

To summarize, this study revealed that in female high anxiety, Low Activity mice, 39 out of the 46 differentially expressed genes associated with oxidative phosphorylation exhibit significant upregulation, identifying important baseline differences in expression of mitochondrial function genes that are both strain and sex specific. In addition to oxidative phosphorylation, several studies have reported anxiety‐related differential gene expression in pathways associated with glycolysis,^59^, ^60^, ^61^ pyruvate oxidation,^43^ oxidative phosphorylation,^60^ and oxidative stress.^60^, ^61^, ^62^, ^63^, ^64^ Together, our RNA‐sequencing study of mice selectively bred for high and low activity adds valuable insights to the growing body of mitochondrial dysfunction and anxiety literature. Moreover, besides Olsen et al.,^63^ our study is unique for the inclusion of female mice.

Future studies should aim to include female mice because anxiety disorders are more prevalent in women^1^ and the majority of differentially expressed oxidative phosphorylation genes in our study were upregulated in female high anxiety, Low Activity mice. As well as the differentially expressed oxidative phosphorylation genes, we also identified 24 genes that appear on our top candidates and two other gene lists previously curated by our lab,^21^, ^25^ highlighting their possible role in activity and anxiety‐like behaviors. In conclusion, mitochondrial dysfunction has recently appeared as both a cause and effect of anxiety‐related disorders and should be further explored as a possible novel pharmaceutical treatment for anxiety disorders.

## Supporting information


**FIGURE S1.** Dendrogram of the hierarchical relationship between the 16 samples depicts clustering by strain, but not sex. Due to the normalization of the Pearson correlation, a smaller number on the *Y*‐axis corresponds to a greater correlation between the samples.

**TABLE S1.** ARRIVE Essential 10 guidelines.

**TABLE S2.** Summary and descriptive statistics for each gene/experimental group and comparisons between groups.

**TABLE S3.** PANTHER enrichment analysis results. We included terms/pathways containing at least 5 gene candidates in the term as candidates, with a Bonferroni adjusted *p*‐value <0.05, and an expected fold enrichment >2. Below are the biological process and molecular function GO terms and PANTHER pathways associated with the three gene lists created from the LRT via DESeq2 (any strain effect, sex‐independent effect, and sex‐dependent effect).

## Data Availability

The data that support the findings of this study are openly available in Gene Expression Omnibus at https://www.ncbi.nlm.nih.gov/geo/, accession code GSE200043.
