# Supplementary material for: Hippocampal RNA sequencing in mice selectively bred for high and low activity
Source: Genes Brain Behav. 2022 Dec 13;22(2):e12832. doi: 10.1111/gbb.12832 (PMC10067415; doi:10.1111/gbb.12832)
Supplement: Supplementary file 1 — FIGURE S1. Dendrogram of the hierarchical relationship between the 16 samples depicts clustering by strain, but not sex. Due to the normalization of the Pearson correlation, a smaller number on the Y‐axis corresponds to a greater correlation between the samples. [file GBB-22-e12832-s001.docx]

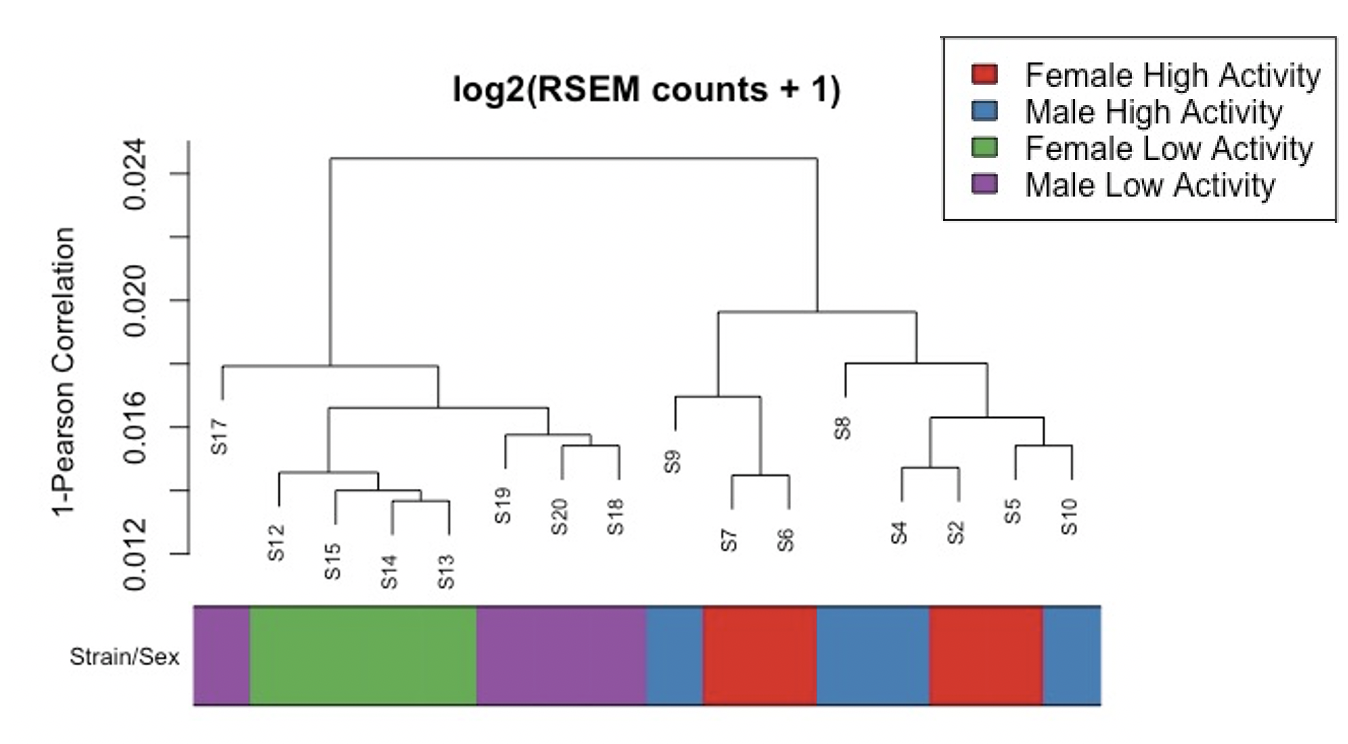


**Supplemental Figure 1.** Dendrogram of the hierarchical relationship between the 16 samples depicts clustering by strain, but not sex. Due to the normalization of the Pearson correlation, a smaller number on the Y-axis corresponds to a greater correlation between the samples.
