# Supplementary material for: Hippocampal RNA sequencing in mice selectively bred for high and low activity
Source: Genes Brain Behav. 2022 Dec 13;22(2):e12832. doi: 10.1111/gbb.12832 (PMC10067415; doi:10.1111/gbb.12832)
Supplement: Supplementary file 4 — TABLE S3. PANTHER enrichment analysis results. We included terms/pathways containing at least 5 gene candidates in the term as candidates, with a Bonferroni adjusted p‐value <0.05, and an expected fold enrichment >2. Below are the biological process and molecular function GO terms and PANTHER pathways associated with the three gene lists created from the LRT via DESeq2 (any strain effect, sex‐independent effect, and sex‐dependent effect). [file GBB-22-e12832-s004.docx]

**Supplementary Table S3.** PANTHER enrichment analysis results. We included terms/pathways containing at least 5 gene candidates in the term as candidates, with a Bonferroni adjusted p-value < 0.05, and an expected fold enrichment > 2. Below are the biological process GO terms, molecular function GO terms and PANTHER pathways associated with the three gene lists created from the LRT via DESeq2 (any strain effect, sex-independent effect, and sex-dependent effect).

| **Any Strain Effect - 3,901 Genes** | | | | |
| --- | --- | --- | --- | --- |
| **Biological process** | | | | |
| **GO term** | **Accession number** | **Associated genes** | **Fold-**  **enrichment** | **Bonferroni adjusted *p*-value** |
| Mitochondrial ATP synthesis coupled electron transport | GO:0042775 | 26 genes | 3.00 | 0.013 |
| Axonal transport | GO:0098930 | 28 genes | 2.76 | 0.024 |
| **Molecular function** | | | | |
| Protein phosphorylated amino acid binding | GO:0045309 | 29 genes | 2.97 | 0.0013 |
| **Sex-independent strain effect - 2,583 Genes** | | | | |
| **Biological process** | | | | |
| **GO term** | **Accession number** | **Associated genes** | **Fold-**  **enrichment** | **Bonferroni adjusted *p*-value** |
| Positive regulation of neuron projection development | GO:0010976 | 51 genes | 2.11 | 0.010 |
| Negative regulation of apoptotic signaling pathway | GO:2001234 | 52 genes | 2.03 | 0.022 |
| **Molecular function** | | | | |
| Protein phosphorylated amino acid binding | GO:0045309 | 22 genes | 3.39 | 0.0035 |
| **Sex-dependent strain effect - 1,318 Genes** | | | | |
| **Biological process** | | | | |
| **GO term** | **Accession number** | **Associated genes** | **Fold-**  **enrichment** | **Bonferroni adjusted *p*-value** |
| Mitochondrial electron transport, NADH to ubiquinone | GO:0006120 | 9 genes | 8.49 | 0.015 |
| ATP synthesis coupled proton transport | GO:0015986 | 10 genes | 8.02 | 0.0068 |
| Mitochondrial respiratory chain complex I assembly | GO:0032981 | 16 genes | 5.83 | 0.00030 |
| Chromatin silencing | GO:0006342 | 18 genes | 4.12 | 0.0068 |
| Axonogenesis | GO:0007409 | 43 genes | 2.20 | 0.022 |
| **Molecular function** | | | | |
| Proton-transporting ATP synthase activity, rotational mechanism | GO:0046933 | 8 genes | 8.02 | 0.027 |
| NADH dehydrogenase (ubiquinone) activity | GO:0008137 | 10 genes | 6.67 | 0.0073 |
| Electron transfer activity | GO:0009055 | 15 genes | 3.88 | 0.035 |
| Structural constituent of ribosome | GO:0003735 | 26 genes | 2.98 | 0.0042 |
| **PANTHER pathway** | | | | |
| Vasopressin synthesis | P04395 | 6 genes | 7.40 | 0.031 |
